# Pathophysiological and Clinical Aspects of Chronic Rhinosinusitis: Current Concepts

**DOI:** 10.3389/falgy.2021.741788

**Published:** 2021-10-27

**Authors:** Stephan Vlaminck, Frederic Acke, Glenis K. Scadding, Bart N. Lambrecht, Philippe Gevaert

**Affiliations:** ^1^Department of Otorhinolaryngology, Centre Hospitalier de Mouscron, Mouscron, Belgium; ^2^Department of Otorhinolaryngology, Ghent University/Ghent University Hospital, Ghent, Belgium; ^3^The Royal National Throat Nose and Ear Hospital, London, United Kingdom; ^4^Laboratory of Immunoregulation, Flemish Institute for Biotechnology, Center for Inflammation Research, Ghent, Belgium; ^5^Department of Internal Medicine and Pediatrics, Ghent University, Ghent, Belgium; ^6^Department of Pulmonary Medicine, Erasmus University Medical Center, Rotterdam, Netherlands

**Keywords:** chronic rhinosinusitis, nasal polyps, eosinophilia, endotyping, allergy

## Abstract

Adult chronic rhinosinusitis (CRS) is a chronic inflammation of the mucosa of the nose and paranasal sinuses. According to the latest EPOS guidelines CRS should be regarded as primary or secondary with distinction between diffuse and localized disease. Further pathophysiologic research identified different inflammatory patterns leading to the term “endotyping of CRS.” The primary focus of endotyping is to define a dominant inflammatory type allowing for better orientation of therapy. The current approach proposes the differentiation between type 2 (eosinophilic) and non-type 2 inflammatory responses. In this review pathophysiological concepts of CRS will be discussed, focusing on the different inflammatory endotypes of T cells with special attention to the eosinophilic type 2 inflammatory response. The contribution of innate and adaptive immune system responses is presented. The possibility of endotyping based on sinonasal secretions sampling is brought to attention because it is indicative of corticosteroid responsiveness and available to most ENT surgeons. Furthermore, the clinical aspects of the three distinct phenotypes are analyzed in view of their characteristics, the related endoscopic findings, typical radiological imaging, histopathology findings, their relation toward allergy and obvious therapeutical implications. This overview will enable clinicians to relate pathophysiological patterns with clinical observations by explaining the different inflammatory mechanisms, hence providing a better understanding of therapy.

## Introduction

Chronic rhinosinusitis (CRS) is a multifactorial inflammatory disease of the nasal and paranasal mucosae presenting with a variety of symptom combinations. Chronic rhinosinusitis may be used to describe conditions ranging from unilateral single sinus disease to widespread sinonasal airway inflammation. The currently recognized definition of primary CRS refers to sinonasal inflammation in which no obvious underlying etiopathogenic event is occurring (i.e., excluding fungal ball, neoplasia, odontogenic or immunodeficiency).

Based on expert recommendations, criteria for CRS were established in the European Position Paper on Rhinosinusitis (EPOS) to sustain uniform epidemiologic studies (1). The EPOS 2012 guidelines describe CRS as an inflammatory disorder defined by the presence of two or more cardinal symptoms [obstruction, drainage (anterior or posterior), smell loss, and facial pain or pressure] for at least 12 weeks duration, confirmed by objective evidence using sinus endoscopy or computed tomography (CT) scan. For study inclusion the guideline requires at least two of four symptoms for at least 3 months duration, one of which must be either nasal obstruction or discharge. According to the new EPOS 2020 classification CRS should now be regarded as primary or secondary, and distinction is made between diffuse and localized disease based on anatomic distribution (2).

Using this definition, epidemiological studies estimated the prevalence of CRS in Europe (10.9%), China (8%), and Brazil (5.5%) (3–5). Studies in the USA, using symptom criteria alone, reported a prevalence of 11.9% resembling the European CRS frequency pattern (6). It is clear however that defining CRS on symptoms alone cannot be sustained as conditions such as odontogenic sinusitis, fungus ball, antrochoanal polyps and others may mimic sustained CRS symptoms and therefore have to be differentiated by adjunctive measures. Recent research showed that epidemiologically defined CRS is not verified by nasendoscopy and CT scan in half the subjects, so the prevalence in Europe is actually 3% if a cut-off score > 4 on the Lund-Mackay scale is used (7). Clinical presentation and CT scanning and/or nasal endoscopy are able to phenotype CRS patients differentiating patients with (CRSwNP) and without nasal polyps (CRSsNP) (8). The European Rhinologic Society and the American Academy of Otolaryngology, Head and Neck Surgery initiated the use of guidelines useful for medical therapy and surgery within a very mechanical understanding of sinus pathology (1, 9). This traditional phenotype-based classification however showed inadequate disease control after medical and surgical treatment probably because it does not mirror the underlying inflammatory disease. Further analysis of this is needed to understand the patient's responsiveness or lack of it to standard treatment.

The recent call for a Precision Medicine Concept now aims for integrated care pathways (ICPs) with treatment protocols adapted to clinical practice (10). Therefore, understanding and identification of different inflammatory types in CRS with proper biomarkers are researched and believed to influence decision making in personalized therapeutic strategies (10, 11). Three phenotypes of primary CRS have been described: allergic, eosinophilic and non-eosinophilic (1, 12). This more pathophysiological view initiated a new term called “endotyping of CRS” and resulted in the search for a more adequate therapy especially for severe and recurrent CRS inflammation (8, 13). An alternative distinction is the inflammatory type dominance, either type 2 (T2) (eosinophilic) or non-T2 (2). T2 can then be subdivided predominantly *via* T helper (Th) 2/allergy/immunoglobulin E (IgE) mechanisms and *via* innate mechanisms (ILCs, innate lymphoid cells) or a mixture of the two (later on in CRSwNP).

The general ENT clinician will be confronted with a subpopulation of Severe Chronic Upper Airway disease (SCUAD) resistant to classic therapy. Some of these individuals will have phenotypes such as aspirin sensitivity, allergic fungal disease or vasculitis, each with specific therapeutic possibilities. Blood tests such as total IgE, IgE, and IgG to Aspergillus and anti-neutrophil cytoplasmic antibodies (ANCA) can aid to identify the latter two, whereas the former requires aspirin challenge testing. Nowadays multidisciplinary teams use molecular knowledge and precision medicine, with close follow-up regarding efficiency and quality control upon novel treatment options, such as biologicals (14). The therapeutic challenge especially applies to the even more complex pediatric SCUAD population in which underlying conditions such as cystic fibrosis and primary ciliary dyskinesia are in the differential diagnosis (15). In the adult population, SCUAD with nasal polyposis and T2 signature is the most challenging phenotype in finding a correct therapeutical rationale combining surgery with potential biologicals. Since these molecules are expensive it makes sense to identify characteristics which identify responders to each particular molecule by submitting data centrally to increase patient numbers (16). To date, this has not happened and the decision-making process for the individual CRS patient is based on careful monitoring of any improvement (17). The complexity of CRS pathology is important as correct medical and/or surgical treatment may largely be beneficial on control improvement of bronchial disease (18). Usefully selected biomarkers are not yet available for predicting a type 1 or type 3 inflammation in CRS; however testing for eosinophils in secretions is simple (19).

Pathophysiological concepts will be discussed in the next chapter, focusing on the different inflammatory endotypes of T cells with special attention to the eosinophilic T2 inflammatory response. This chapter is followed by an overview of the three main inflammatory sinonasal phenotypes, focusing on diffuse disease.

## Pathophysiology

To clarify pathophysiological aspects of CRS, some general immunological topics need to be known. Lymphocytes play an important role in the innate and adaptive immune system. Two major types can be distinguished: B lymphocytes originating from the bone marrow and T lymphocytes arising from the thymus. In the adaptive immune response, T cells are generated in the secondary lymphatic tissues to encounter antigens and become antigen-specific cells after proliferation. B cells, after being influenced by T cells, become antibody-secreting cells. Natural killer (NK) cells, activated by interferons (IFNs), are a third type belonging to the innate immune system and recognize changes in the major histocompatibility complex (MHC) class 1 (20). The main focus regarding CRS pathophysiology is on T cells.

### T-Cell Physiology

**Figure 1:**
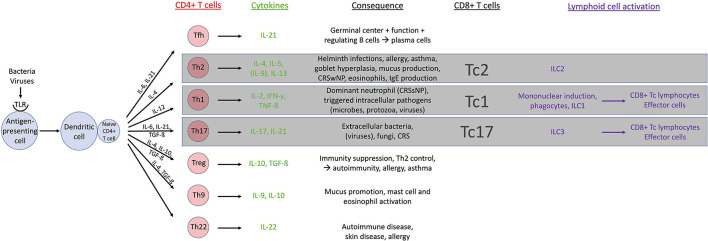
Overview of T cell physiology. CD, cluster of differentiation; CRS, chronic rhinosinusitis; CRSsNP, CRS without nasal polyps; CRSwNP, CRS with nasal polyps; IFN, interferon; IgE, immunoglobulin E; IL, interleukin; ILC, innate lymphoid cell; Tc, cytotoxic T cell; Tfh, T follicular helper cell; TGF, tumor growth factor; Th, T helper cell; TLR, Toll like receptor; TNF, tumor necrosis factor; Treg, regulatory T cell.

T lymphocytes and their cytokines influence the cell-mediated immune response through activation *via* the T cell receptor (TCR) and the co-stimulatory molecule cluster of differentiation (CD) 28. Activation results in the production of interleukin (IL)-4 and IL-10 facilitating T-cell/B-cell interaction. T lymphocytes can be immunophenotyped in CD3+CD4+ and CD3+CD8+ white blood cells by their cell surface identification molecules. CD8+ cells, also known as cytotoxic T (Tc) cells, recognize MHC-1 molecules on the surface of infected cells and are bound to eliminate those. CD4+ cells, also known as Th cells, recognize MHC-2 molecules on the outer layer of antigen-presenting cells (B cells, macrophages and dendritic cells). When CD4+ T cells are stimulated by an antigen, further differentiation occurs with different cytokine patterns and distinct cellular function *in vivo*: actual importance is retained in Th1, Th2, Th17, regulatory T (Treg), and T follicular helper (Tfh) cells (21) (Figure 1).

### Different Types of Immune Responses

Immune polarization is based on T cell cytokine production. The emerging linkage between adaptive and innate immune systems has led to the proposal of type 1, 2, and 3 immune responses (22). Type 1 immune responses are characterized by type 1 innate lymphoid cells (ILC1), and Tc1 and Th1 cells. The crucial role of this type 1 immune response is to deal with intracellular microbes, protozoa and viruses (23). The activation of ILC1, Tc1, and Th1 cells will induce the production of type 1 cytokines IFN-γ and tumor necrosis factor (TNF)-α resulting in the activation of mononuclear phagocytes loaded with potent cytotoxic molecules (22). Type 2 immune responses implicate ILC2s, Tc2, and Th2 cells responsible for the production of IL-4, IL-5, and IL-13 cytokines. Type 2 plays an important role in parasite infection and induces allergic diseases with important contribution of eosinophilic cells, IgE production and goblet cell hyperplasia (22, 24). Type 3 immune responses are currently associated with cytokines IL-17 and IL-22 and controlled by IL-3, Tc17 cells, and Th17 cells. Their role is believed to facilitate immune responses opposing extracellular bacteria and fungi (22). As an overview, CD4+ (Th) and CD8+ (Tc) cells with their cytokines in CRS are described below.

**Th1 cells:** these cells are mainly activated by intracellular pathogens. Bacterial and viral products are bound to Toll like receptors (TLR) on antigen presenting cells (APC). Hence dendritic cells will secrete IL-12 cytokines leading to the production of typical Th1 cytokines: IL-2, IFN-γ, and TNF-ß expressing a dominant neutrophil pattern.

**Th2 cells:** during a type 2 immune response Th2 cells produce key cytokines IL-4, IL-5, IL-9, and IL-13, which induce antibody class switching to immunoglobulin (Ig)E and IgG1, and enhance the recruitment of inflammatory cells (predominantly eosinophils, basophils and mast cells). Goblet cell hyperplasia is stimulated and mucus production is induced. Th2 response is essential to fight parasitic infections, but also promotes allergic disease and asthma.

**Th17 cells:** implication of Th17 cells is considered as an immediate response to extracellular bacteria and fungi. The production of IL-17 and IL-22 cytokines may cause chronic inflammatory disease and autoimmune pathology when dysregulation is present.

**Tfh cells:** Tfh cells are recognized important in regulating B cells to support antibody response. IL-21 is considered the signature cytokine.

**Treg cells:** the two cytokines mainly associated with Tregs are IL-10 and tumor growth factor (TGF)-β. Tregs secrete these cytokines and use them to carry out a suppressive function on the immune system. It has shown importance in controlling Th2 responses. Lacking those cells may allow further development of asthma and allergy, as well as autoimmune diseases (25).

**Th9 cells:** IL-9 has been identified in a subset of T cells distinct from Th2 cells. The production of IL-9 requires the combination of TGF-β (which also promotes Tregs) and IL-4 (known to induce Th2 cells). Interestingly, Th9 cells, which are strongly associated with the immunopathology of asthma, also produce IL-10. IL-9 seems important in promoting mucus production and activation of mast cells as well as eosinophils (25).

**Th22 cells:** Th22 cells represent a recent separate Th subset and are closely related to Th17 cells. They predominantly produce the cytokine IL-22 and were initially associated with immunopathology of skin diseases. Recent evidence indicates that IL-22 plays an important role in the pathogenesis of autoimmune diseases and allergic diseases (25).

**CD8+** **cells:** The CD8+ Tc lymphocytes seem to mirror the Th cell subset classification based on their transcription factor and cytokine expression patterns forming counterparts toward the CD4+ cell line namely Tc1, Tc2 and Tc17 cells (24, 26).

A large heterogeneity in CRS immune polarization is seen worldwide as the immune responses vary across different geographic areas and populations with distinct racial backgrounds (27–30). Immune responses toward type 1, type 2, and type 3 directions can define certain endotypes and may therefore influence clinical manifestations of CRS pathology (31). CRSwNP and CRSsNP nowadays may be linked to inflammatory patterns associated with Th1 (type 1), Th2 (type 2), or Th17 (type 3). CRSsNP is accepted to exhibit a type 1 immune response (28, 29). In Europe, Caucasian patients with CRSwNP mainly demonstrate a type 2 immune response with high asthma comorbidity (1, 32, 33), whereas in China and other East Asian countries, patients with CRSwNP show ~50% less type 2 cytokine expression with less eosinophilic inflammation and lower asthma comorbidity (34). Asian patients with CRSwNP predominantly show neutrophil-biased inflammatory patterns (34). The association of type 2 immune responses with the development of nasal polyps is sustained in a clustering analysis of CRSsNP vs. CRSwNP in Caucasian patients (35).

### Type 2 Immune Responses and CRS

**Figure 2:**
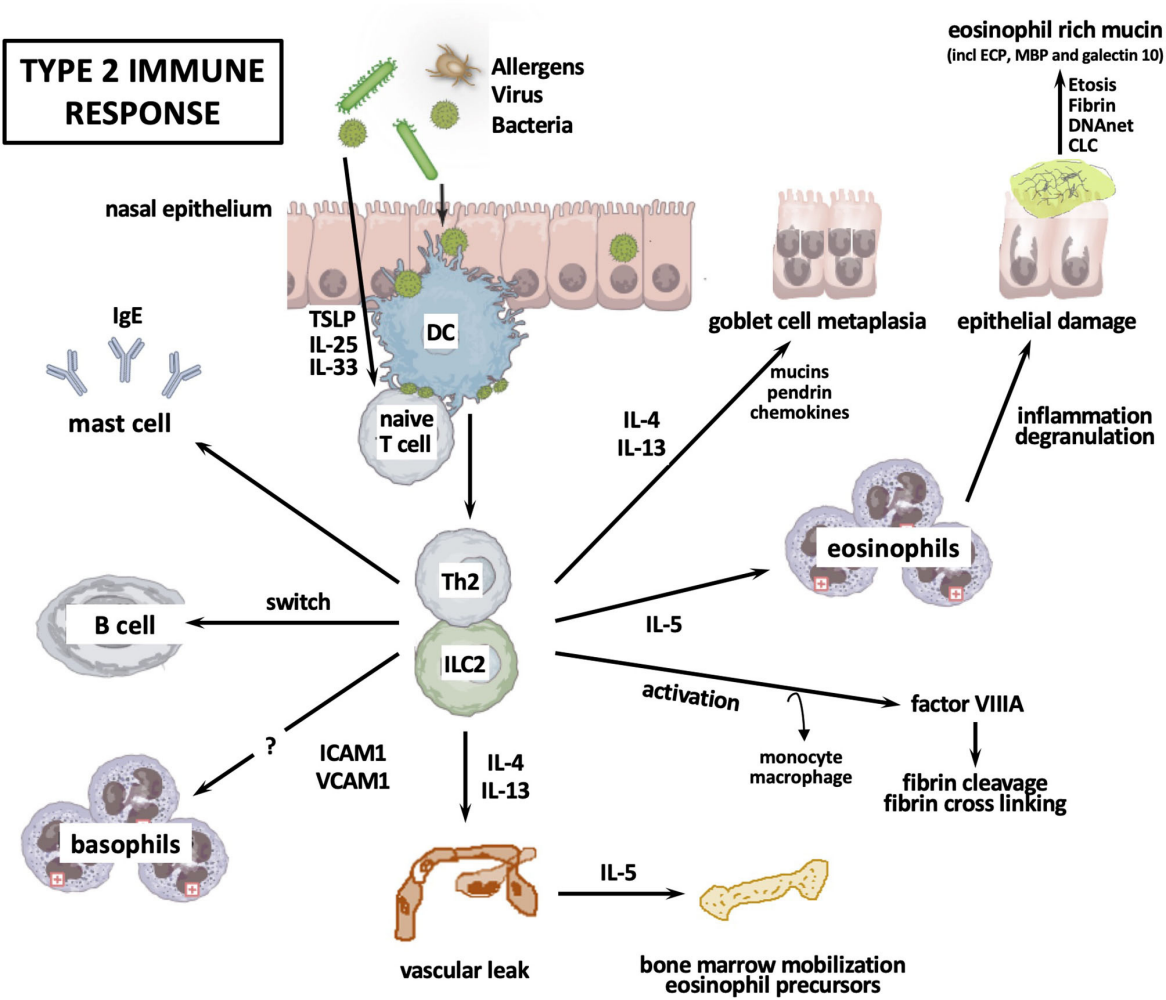
Type 2 immune response. CLC, Charcot-Leyden crystals; DC, dendritic cell; ETosis, eosinophil apoptosis; ICAM, intercellular adhesion molecule; Ig, immunoglobulin; IL, interleukin; ILC, innate lymphoid cell; Th, T helper cell; TSLP, thymic stromal lymphopoietin; VCAM, vascular cell adhesion molecule.

Most European Caucasian and some Asian CRSwNP patients show increased numbers of Th2 and Tc2 cells, which could be associated with mucosal eosinophilia (34, 36). This type 2 immune response in CRSwNP is supported by the elevation of ILC2, the increased presence of tissue eosinophilia, a clear upregulation of IL-4, IL-5, IL-13, and local IgE, and profound tissue eosinophilia independent of atopy (37) (Figure 2).

After stimulation with innate immune-activating stimuli, cytokines, or injurious environmental agents such as proteases, epithelial cells produce thymic stromal lymphopoietin (TSLP) and sometimes IL-33 or IL-25, which activate ILC2. One example is the induction of IL-13 by IL-33 in reaction to the protease activity of Aspergillus Fumigatus (38). Epithelial cell-derived TSLP upregulates OX40 ligand (OX40L) expression on dendritic cells, and then dendritic cells initiate the differentiation of naive T cells into Th2 cells. Th2 cells, ILC2, and Tc2 cells orchestrate eosinophilic inflammation through production of type 2 cytokines. IL-4+ IL-21+ Tfh cells initiate the differentiation of B cells into plasma cells, followed by mast cells activation due to IgE, which is locally produced by plasma cells. Subsequently, mast cells can produce type 2 cytokines. Th2 inflammation can also induce monocytes and macrophage differentiation into M2 macrophages. M2 macrophages produce coagulation factor XIII-A (FXIII-A) that induces excessive fibrin deposition by cross-linking of fibrin and by antifibrinolytic pathways through binding the α2-plasmin inhibitor (α2-PI, also known as α2 antiplasmin) to fibrin (39). Meanwhile, tissue plasminogen activator (t-PA) levels are lowered in Th2 inflammation, causing impaired plasmin generation, which in turn decreases fibrinolysis (40). These events collectively result in the retention of water and the formation of edema in polyps. Th2 cytokine-mediated pendrin expression can increase mucus production. Cytokines IL-4 and IL-13 can decrease the expression of epithelial cell tight junction proteins. Neutrophil-derived oncostatin M (OSM) and eosinophil-derived DNA traps can also contribute to epithelium disruption.

Typical for the type 2 immune response is the increased production of local IgE in association with mucosal eosinophilia (33, 41), as well as the increased mucosal infiltration of B cells with the presence of markers of class switch recombination to IgE in CRSwNP patients (41–43). Of interest, Tfh cells may be found in germinal centers in secondary lymphoid tissue and are important to generate B cell responses. This is supported by the finding of ectopic lymphoid tissue in nasal polyps and the finding of Tfh cells in loco (44).

Literature reports Staphylococcus Aureus enterotoxins to act as antigen and superantigen inducing local IgE production (33). This could not be confirmed in the analysis of Chinese patients with eosinophilic CRSwNP (41). Polyclonal IgE antibodies have been shown to activate mast cells in nasal polyps (45–48) and IgE-mediated mast cell activation is found to be upregulated in eosinophilic nasal polyps (49). The finding of elevated infiltration of basophils in tissue of eosinophilic CRSwNP remains to be analyzed (50).

### Type 1 and 3 Immune Responses in CRS

The type 1 immune response of CRSsNP expresses IFN-γ cytokines in Caucasian patients (28, 29). Asian patients and patients with cystic fibrosis-related nasal polyps present with a neutrophil-related inflammation with high levels of IFN-γ and IL-17A expression (12), the latter pointing toward a type 3 immune response. The IFN-γ upregulation could however not be retained in a CRSsNP Chicago study suggesting a geographical variation (51).

**Figure 3:**
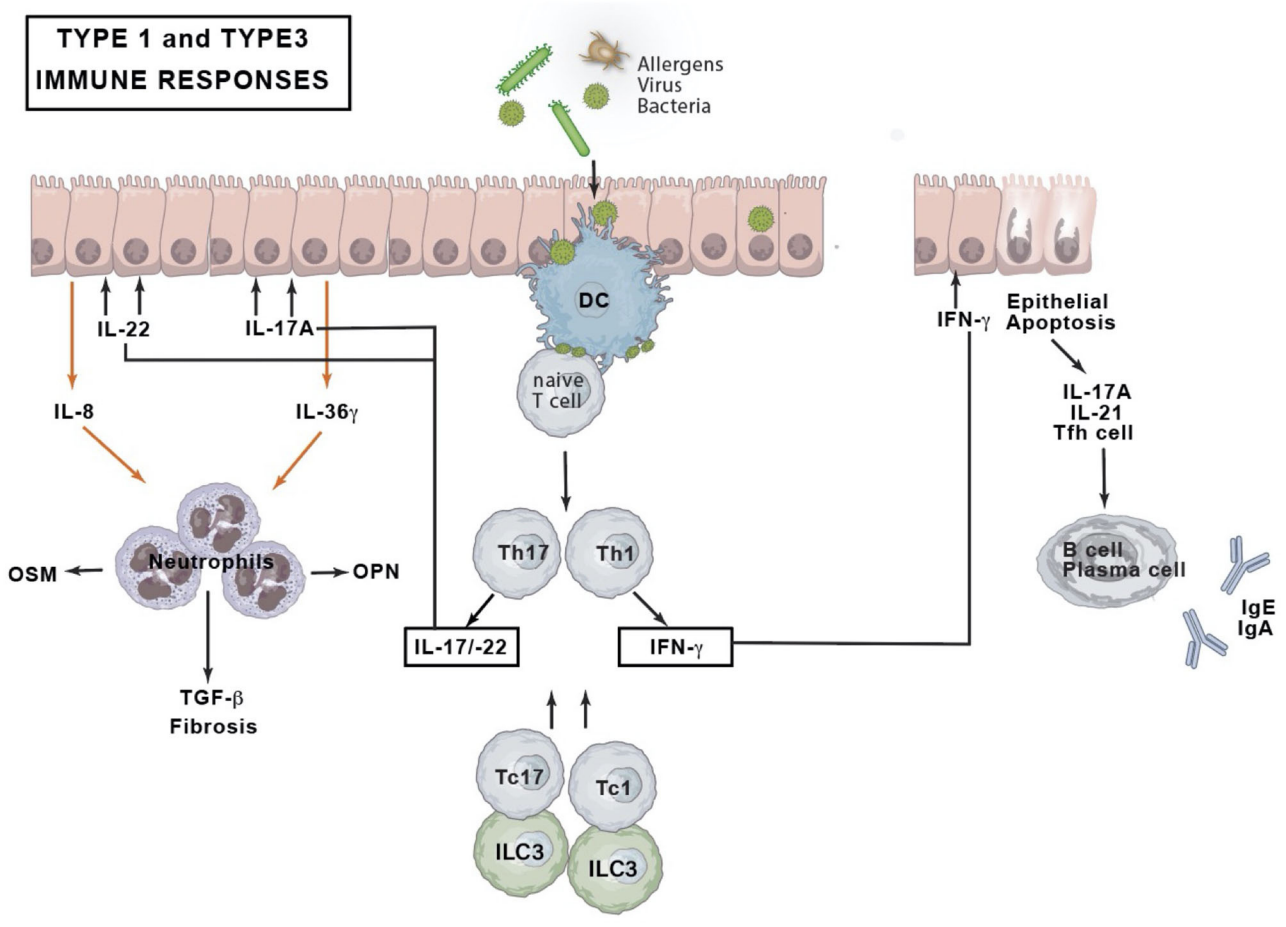
Type 1 and type 3 immune response. DC, dendritic cell; IFN, interferon; Ig, immunoglobulin; IL, interleukin; ILC, innate lymphoid cell; OPN, osteopontin; OSM, oncostatin M; Tc, cytotoxic T cell; Tfh, T follicular helper cell; TGF, tumor growth factor; Th, T helper cell.

In type 1 and type 3 immune responses environmental triggers will stimulate epithelial secretion of osteopontin (OPN) hence triggering dendritic cells to activate Th1 and Th17 cells (52) (Figure 3). Together with Tc1 and Tc17, Th1, and Th17 cells orchestrate non-eosinophilic inflammation through production of IFN-γ, IL-17A, and IL-22 (31). IFN-γ induces apoptosis of epithelial nasal cells, disrupts tight junctions and stimulates neutrophils phagocytosis and chemotaxis (52, 53). IL-17A upregulates the expression of IL-36γ in epithelial cells, whereas the latter acts on neutrophils and further exaggerates neutrophilic inflammation by inducing IL-8 [C-X-C chemokine ligand-8 (CXCL8)] production from neutrophils (54). IL-22 induces epithelial cells to produce IL-8/CXCL8, which also acts on neutrophils. Neutrophils might produce OSM, OPN, and TGF-β2. TGF-β2 is supposed to be involved in fibrosis. IFN-γ and OSM could disturb epithelial barrier function by decreasing the expression of epithelial cell tight junction proteins. IFN-γ can induce activated but insufficient autophagy, leading to apoptosis of nasal epithelial cells. IL-17A+ IL-21+ Tfh cells initiate B cell differentiation into plasma cells that produce immunoglobulins G and A (IgG and IgA).

### Endotyping CRS – Role for Mucus Sampling

The variety in pathophysiological mechanisms of CRS makes the achievement of a universal analysis regarding clinical characteristics, molecular biomarkers, genetics, histopathology, epidemiology and treatment response difficult. Of interest, although on a small number of patients (*n* = 32), a simplified commercially available 41-plex cytokine-chemokine array on sinonasal tissue allowed the detection of three distinct endotype signatures helpful to decide on individualized therapy (35). To date, most studies are in search of biomarkers based on tissue sampling obtained during invasive procedures (41). A more feasible and possible repetitive approach involves the sampling of mucus (55). In the cluster analysis of the latter study, inflammatory mediators pointing toward types 1, 2, and 3 inflammatory patterns were consistent with results based on tissue analysis (55). Even the presence of the Th2-associated cytokines IL-5 and IL-13 could recently be detected in sinonasal secretions by multiplex cytometric assay, suggesting possible early stratification of CRS subgroups and more personalized therapies (55). Cellular analysis of sinonasal secretions in CRSwNP patients more often shows a Th2 bound inflammation whereas the presence of eosinophil-rich mucin may be considered an easy-to-obtain biomarker for predicting recurrence of CRSwNP with higher need for surgery and for predicting asthma development (56). Recent research highlights the importance of Charcot-Leyden crystals (CLC) as a relevant Th2 marker within CRS secretions and its implications on their finding in sinonasal secretions of CRSwNP patients and in impacted bronchial secretions of eosinophilic asthma patients, opening new targets for therapeuticals (57).

In analogy with sampling of sinonasal secretions Seys et al. clearly could demonstrate in asthmatic sputum a diversity of type 2 cytokines discerning also non-type 2 cytokines concluding a priori the likeliness of type 2 vs. non-type 2 molecular asthmatic phenotypes (58).

## Clinical Presentation

**Figure 4:**
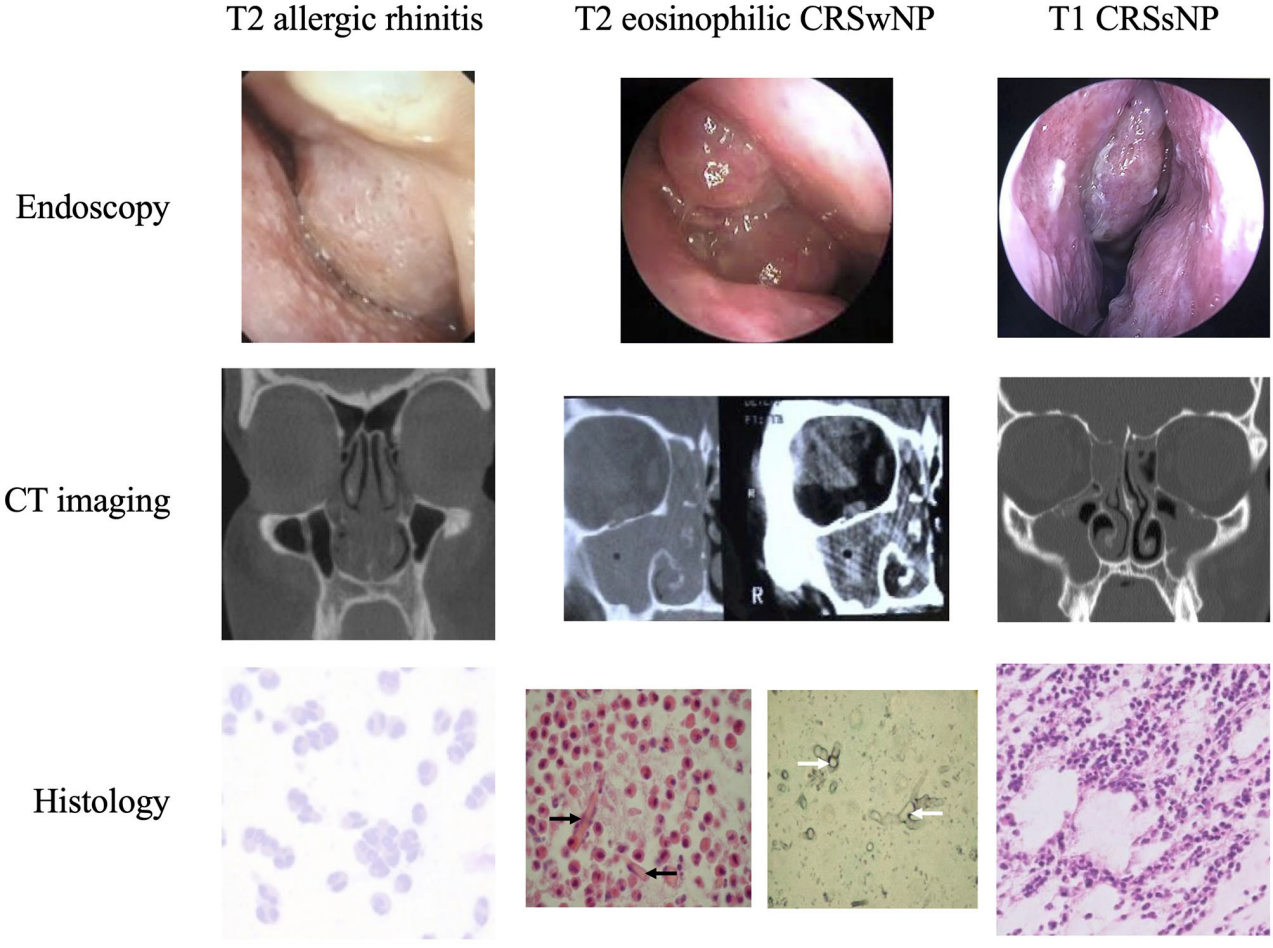
Typical examples of endoscopic, CT and histologic (sinonasal secretions) images of the three main inflammatory sinonasal phenotypes. Images of type 2 (T2) allergic rhinitis show congestion of the nasal turbinates, normal aeration of the paranasal sinuses and mainly eosinophilic cells on a nasal smear. Images of T2 eosinophilic chronic rhinosinusitis with nasal polyps (CRSwNP) show the presence of nasal polyps on endoscopy, opacification of the paranasal sinuses on CT bone window and a hyperattenuation pattern on CT soft tissue setting. Corresponding histology findings are eosinophilic and necrotic patterns with Charcot-Leyden crystals (latter shown by black arrows) on hematoxilin/eosin staining, and fungal hyphae (white arrows) on silver staining. Images of T1 chronic rhinosinusitis without nasal polyps (CRSsNP) show opacification of the paranasal sinuses on CT and a mainly neutrophilic content in the nasal secretions.

Inflammatory sinonasal disease may be grossly divided in eosinophilic airway inflammation vs. non- eosinophilic inflammation. Three endotypes show a distinct T2 eosinophilic airway inflammation namely allergy, eosinophilic CRS and CRSwNP vs. a non-eosinophilic T1 inflammation pattern also present in the CRSsNP and some of the CRSwNP population. Those inflammatory patterns will be described below in addition to the concept of united airways. An overview of the characteristics of these endotypes is provided in Table 1, whereas endoscopic, CT and histologic (sinonasal secretions) images are shown in Figure 4.

**Table 1:** Overview of the three main inflammatory sinonasal phenotypes with their characteristics (CRS, chronic rhinosinusitis).

**Phenotype**	**Allergic rhinitis**	**Eosinophilic CRS**	**Non-eosinophilic CRS**
Type of secretions	Watery secretions	Thick tenacious mucin	Discolored secretions
Appearance of eosinophilic cells	Intact eosinophilic cells	Necrotic eosinophilic cells (ETosis)	Mainly neutrophilic cells (NTosis)
Charcot-Leyden crystals (CLC)	No CLC	CLC present	No CLC
Appearance of granules	No granules, no proteins	Granule proteins	Free eosinophilic granules
Type of cytokines	T2 cytokines	T2 cytokines	T1 cytokines
Typical age of patients	Likely young population	Likely older population	Diverse
IgE involvement	Evidence of IgE-mediated	IgE not necessarily present	IgE not likely
Presence of nasal polyps	Nasal polyposis not likely	Nasal polyposis likely	(Small) nasal polyps possible
State of mucosal lining	No damage of mucosal lining	Possible mucosal damage	Possible mucosal damage
Presence of hyphae	No hyphae	Hyphae possible	Hyphae not likely
Presence of major basic protein (MBP)	No MBP	MBP present	MBP not likely
CT appearance	Typical black halo on CT	Possible CT hyperattenuation	Atypical sinusitis on CT
Presence of asthma	Asthma with early onset	Late-onset asthma, eosinophilic	Atypical asthma
Possibility of oral steroids	Oral steroids rarely	Oral steroids more frequently	Oral steroids rarely
Possibility of vaccination	Possible vaccination	Vaccination rarely	No vaccination considered
Standard oral therapy	Anti-allergic therapy	Steroids, monoclonal antibodies	Antibiotics
Typical evolution	Tendency to disappear with age	Tendency to aggravate with age	Aggravation with age (multifactorial)
Persistence of disease	Restricted lifetime pathology	Lifetime pathology	Multifactorial dependent lifetime

Eosinophilic airway inflammation includes allergic disease such as allergic rhinitis in which the sinuses are usually minimally involved. Eosinophilic airway inflammation is also noted in CRSsNP and CRSwNP subdivisions. On the other hand we face non-eosinophilic airway inflammation in CRSsNP and in some CRSwNP subgroups.

The impact of upper airway inflammation on the lower airways is currently investigated for its impact by collecting real-life data and confirms the high disease burden in uncontrolled CRS patients, clearly impacting quality of life. Mobile technology such as Galenus Health opens a new era of real-life monitoring giving valuable clinical information about the relationship between upper and lower airways (59).

### Allergic Airway Inflammation

#### Defining Characteristics

Patients with allergic rhinitis (AR) often show an earlier onset of disease, namely at younger age (<20), and although there is eosinophilic Th2 cell involvement, the disease is mainly immunoglobulin E (IgE) driven with other signs of atopic disease. Local symptoms are more dominated by itch, sneeze, and watery rhinorrhea. The presence of hyposmia rather suggests chronic rhinosinusitis with or without nasal polyps than rhinitis (60). The symptoms remain corticosteroid responsive and might soften and disappear with age (61). There is only weak evidence supporting a connection between CRS with/without nasal polyps vs. an allergic CRS condition (62). Since sinus involvement is minimal, allergic airway inflammation should probably not be included as part of CRS but be re-designated Persistent Polypoid Allergic Rhinitis (63).

#### Endoscopy

In patients with AR inhaled allergens are deposited on the head of the middle turbinate with possible inflammation and edema of the mucosa. The middle turbinate edema in more advanced cases can extend to the superior turbinate and posterior nasal septum and narrow or obstruct the more lateral sinus ostia (64). However, the presence of thick eosinophilic mucin as seen in eosinophilic CRS patients is by far less common in this allergic phenotype. Even with extreme polypoid change, there is often near normal ethmoid, sphenoid and maxillary mucosa, and simple trapped mucus is mostly found at surgery (65).

#### Radiology

The typical “black halo” sign originally described by Lund et al. shows a central thickening of the turbinates and septum with near normal peripheral sinus mucosa and is considered typical for inhalant/IgE driven CRS (66).

#### Histopathology

On histopathology T2 cytokines dominate and elevated total and serum specific IgE is found. Elevated serum eosinophil count is only rarely observed and tissue sampling is performed with simple hematoxylin and eosin (H&E) coloring (61). Of most importance, eosinophilic mucin and CLC are not found in this condition as being typical to eosinophilic CRS conditions.

#### Allergy

Patients with AR show higher serum specific IgE compared to other subtypes of asthma (61). A Positive skin prick test or Immunocap/radioallergosorbent test (RAST) sustains the diagnosis. Differentiation has to be made between perennial and seasonal allergic disease when therapy is required (67).

#### Therapeutic Implications

Current treatment guidelines provided by the Allergic Rhinitis and its Impact on Asthma (ARIA) Task Force propose a stepwise approach with medical treatment and when intractable disease is faced, immunotherapy has to be considered. Only in extreme conditions of tissue remodeling adjunctive surgery might be considered as an ultimate step (64, 67).

### Eosinophilic Airway Inflammation

#### Defining Characteristics

Eosinophilic upper airway pathology is an inflammatory disease based on a T2 response driven by an eosinophilic inflammation. Most of those patients show an adult-onset history mainly from 30 toward 50 years of age (68). This condition is mostly found in CRS patients with nasal polyps (CRSwNP) and is often characterized by eosinophilic inflammation with elevated levels of T2 cytokines (69, 70). The tissue eosinophilia in CRSwNP is frequently associated with extensive sinus disease (71), higher post-operative symptom scores (72), less improvement in both disease-specific and general quality of life (73), and a higher polyp recurrence rate (74–76). Moreover, CRSwNP patients with type 2 inflammation show a higher risk of late-onset asthma comorbidities, multimorbidity and recurrence of disease after surgery (75, 76). Noteworthy, a small number of CRS patients without nasal polyps (CRSsNP) may show a T2 signature and yield tissue and sinonasal secretions with eosinophilic infiltration (76). It is not clear if this subgroup holds the initial phase toward the development of nasal polyps. CRSwNP must be differentiated from other nasal polyp conditions like nasal choanal polyps, inverting papilloma and cystic fibrosis.

#### Endoscopy

At endoscopy patients will frequently present with small or already larger polyps typically protruding from the middle meatus. When attention is given the finding of tenacious eosinophil mucin may be encountered and collected on simple aspiration on consultation or at surgery. This eosinophil mucin helps further identification of a T2 bound inflammation on histopathology.

#### Radiology

Very often those patients show a pan-sinus opacification with evidence of secondary obstruction on CT imaging. Neo-osteogenesis changes are common even in the non-operated patient. On CT soft window imaging a central density in sinuses may be present compatible with thick mucin. On T2 weighted magnetic resonance imaging (MRI) those densities may appear as a signal void (77).

#### Histopathology

High eosinophilic blood levels yield a positive likelihood ratio (LR) of 3.28 to predict high tissue eosinophilia but the latter is not significantly associated with serum allergen specific IgE (78). Few studies have investigated the level of mucosal eosinophil density required to meet the definition of tissue eosinophilia. Various eosinophil numbers per high power field (HPF) were used in different studies with cutoff values ranging from 5 to 350 eosinophils/HPF. A recent systematic review showed a cut off 55 eosinophils/HPF was likely to predict recurrence following surgical intervention (79). The existence of geographic, ethnic, and environmental differences suggest that specific cutoff values may be considered in different populations and regions (80). The Japanese Epidemiological Survey of Refractory eosinophilic CRS presented a new algorithm with diagnostic criteria based on scores comprising bilateral disease sites, nasal polyps, CT findings, and eosinophilia in peripheral blood. Reaching a score of 11 points was considered diagnostic for eosinophilic CRS. Significancy was reached with a cut-off value of more than 10% blood eosinophilia and tissue analysis with 70 eosinophils/HPF. Though the Japanese proportion is almost equal to that observed in Western countries <50% of polyps in Asian patients show tissue eosinophilia (81).

The analysis of sinonasal secretions is of growing interest, as these are easy to obtain in contrast with tissue sampling. Occurrence of eosinophil apoptosis (ETosis) is shown by the presence of eosinophilic free vesicles containing toxic proteins but also by the presence of CLC as a T2 eosinophilic hallmark (82). Recently, the finding of eosinophil rich mucin (ERM) in patients with T2 bound CRSwNP was proven to be a predictor for NP recurrence after surgery, the need for revision surgery and appearance of late onset asthma (56). Very recently the importance of CLC in secretions has been stressed by mouse models and the analysis and research aiming at the dissolution of crystals opens new horizons in human treatment facilities of pulmonary and sinonasal T2 inflammatory disease (57, 83).

#### Allergy

A large number of these patients show no allergy at all whereas in others IgE sensitization or even a multiallergen sensitization can be found. Mechanisms with local mucosal IgE generation inducing multi-allergen sensitivity have been described (33). Staphylococcus aureus might act as a superantigen in difficult to treat patients with nasal polyps and concomitant asthma (84). CRS patients exhibiting evident allergic reaction to fungi (e.g., a positive skin prick test and/or elevated specific IgE) can still be named allergic fungal rhinosinusitis (AFRS) as this term is commonly used according to EPOS 2020 (2). AFRS is considered a clinical subtype of CRSwNP based on an innate type 2 immune response. It is characterized by the presence of eosinophilic mucin with non-invasive fungal elements; typical imaging signs of CT hyperdensity and signal void on T2 MRI images may be present. The controversy is directed at the presence of at least one positive IgE-mediated allergy to one or more fungi. This IgE-bound inflammation rises the assumption of a possibly different endotype. Treatment is based on oral steroids and surgery with debridement of the sinuses. Good rationale exists for the use of biological agents targeting the eosinophilic inflammation or other type 2 responses (85–87).

#### Therapeutic Implications

Medical management has been outlined in the EPOS guidelines in a stepwise approach targeting the disease severity (1). The goal is to deliver anti-inflammatory medicine to the site of the disease with the least amount of side effects or systemic exposure. When exacerbations occur with limited burden of disease, intermittent short courses (2–3 weeks) of glucocorticosteroids (GCS) can be offered 2–3 times per year. Although medical management with intranasal and oral glucocorticosteroids has been shown to be effective in mild cases, the side effects of long-term use of GCS urges the need for surgical intervention (88). Some medical treatments of NP patients based on glucocorticoids and doxycyclin therapy show temporary success (89, 90).

Sinus surgery is performed in an attempt to control disease and improve the patients' symptoms and overall quality of life. Proposed surgical techniques vary from the least extensive polyp extraction to the most extensive nasalization procedures (91, 92). Due to the high recurrence rate in CRSwNP patients a tendency toward more extended approaches have been proposed for better access to the sinuses for more adequate local treatment and reducing the inflammatory load (91, 93). After the surgical creation of large open cavities the delivery of high-volume glucocorticoid nasal irrigations showed to be more effective vs. nasal spray in preventing endoscopic evidence of recurrence (94). Over decades, performing endoscopic surgery, it was generally accepted that stripping of the mucosa was to be avoided fearing scarring, chronic osteitis and non-functional mucosa (88). Nowadays a new concept is arising called reboot surgery based on the removal of all inflamed sinus mucosa for type 2 inflammatory CRSwNP (95).

Other therapeutic considerations are necessary as a significant number of patients continue to have upper and lower airway symptoms despite classic medical and surgical treatment. Humanized and fully human monoclonal antibodies (mAbs) such as anti-IgE, IL-5, and anti-IL-4 receptor α are increasingly used (96–98). Not reaching a 100% success rate and the high cost for long-term treatment urge the need for alternative products. Evaluation of biological treatments in CRSsNP patients with signs of type 2 inflammation will be crucial in the development, together with the further search for biomarkers to identify responders to those treatments (99).

The sinonasal outcome test (SNOT)-22 score is recommended as a useful tool in symptom severity scoring by also evaluating emotional and social consequences of the condition. The objectification of eventual individual improvement by medical therapy and/or surgery can be interesting for study purposes as well as in evaluating quality of life (QoL) repercussions (100).

### Non-eosinophilic Airway Inflammation

#### Defining Characteristics

Patients with a non-eosinophilic airway inflammation may be considered as non-type 2 and are mainly characterized by neutrophils in their nasal mucosa (31, 70). These conditions are present in infectious rhinitis, CRSsNP and the Th17 pathway currently addressed now as type 3 immune response. In Asia, non-eosinophilic CRSwNP is frequently observed and is associated with relatively less edema and more fibrosis compared with eosinophilic CRSwNP (50). Of note, the presence of a mixed Th17/Th2 inflammation in CRSwNP is possible as neutrophil-biased inflammation may be demonstrated in eosinophilic nasal polyps. The combination of high levels of type 2 inflammation mediators combined with high levels of type 1 or type 3 and/or neutrophilic markers seems to predict a more severe inflammatory burden (33). Neutrophilic inflammation can be triggered by infections or chronic irritation, environmental toxins, work conditions and air pollution. Very often tissue neutrophilia is not completely controlled by inhaled GCS (101, 102).

#### Endoscopy

Small polyps and polypoid edema may be seen in these patients. The aspect will rather show inspissated secretions but not like eosinophil mucin. Those thick discolored secretions can obstruct the sinus outflow tracts and cause retro-obstructive retention in the paranasal sinuses with purulent postnasal drip patterns.

#### Radiology

This type of neutrophilic inflammation cannot be distinguished from imaging of the eosinophilic type as sinuses can be diffusely involved.

#### Histopathology

Tissue neutrophilia is significantly higher although some eosinophils may be present. The presence of the non-T2 cytokines in the mucus is correlated with higher culture positivity and age (103). The analysis of sinonasal secretions may contribute to depicting the presence of an active neutrophil extracellular trap (NET) mechanism with the release of chromatin (NTosis) and granule proteins that bind and kill microorganisms (104–106). In contrast ETosis depicts typical free eosinophil granules (FEG) with regulated release of toxic proteins such as major basic protein (MBP) (107).

#### Allergy

Overall patients will have a negative skin prick and immunocap/RAST testing, and poor clinical evidence of allergen driven symptoms.

#### Therapeutic Implications

When medical therapy fails patients will benefit from having sinus surgery allowing for saline washings and local application of medical therapy. This type of patients may benefit from long-term low dose macrolide immunomodulation especially CRS patients with limited response to corticosteroids (108, 109).

### United Airways

The united airway hypothesis links the entire upper and lower airways as an interconnected system sharing the same inflammatory responses. Although Brown demonstrated the importance of sputum eosinophils in relation to corticosteroid responsiveness in asthma in 1958 (110), the concept of asthma being heterogeneous has only recently gained traction with the advent of biologicals. The term “asthma” is currently considered an umbrella diagnosis for different disorders (endotypes) and phenotypes (e.g., allergic, obesity associated, aspirin-sensitive, fungal allergic, and elderly). It is characterized by reversible airflow obstruction and its main symptoms include wheezing, shortness of breath, cough and chest tightness (111). Asthma endotypes may be broadly regarded as type 2 high or T2-low (111), similarly to CRS. In this way, it follows the heterogeneity of chronic rhinosinusitis which has been known for a long time, probably because of ease of access of the upper airways for examination and investigation.

Most Western asthma patients (78%) have some form of upper airway disease, with similar levels of severity (112). In return, in a recent UK analysis the prevalence of asthma increased from control participants (10%) over CRSsNP (21%) to CRSwNP (47%) and AFRS (73%) (113). Typical associations are pollen asthma together with seasonal allergic rhinitis, and persistent rhinitis and chronic asthma. In one study 84% of severe asthma patients had sinonasal CT abnormalities. The latter correlated with eosinophils in peripheral blood and induced sputum, and with the level of exhaled NO. Sinonasal CT scores also related to lung function measurements: positively to functional residual capacity and inversely to diffusion capacity (114). Western eosinophilic CRS patients, with and without nasal polyps, frequently have asthma with shared histological and immunological features, characterized by an environment high in T2 cytokines (IL-4, IL-5, and IL-13) and in ILC2s (27, 115), suggesting a common immune process involving the upper and lower airways (116, 117). In contrast, T2-low asthma is characterized by neutrophilic (sputum neutrophils >40–60%) or paucigranulocytic (i.e., normal sputum levels of both eosinophils and neutrophils) inflammation and a lack of response to corticosteroid therapy. It has been linked to Th1 and/or Th17 cell activation and their imbalance may play a role in steroid-resistant, severe and neutrophilic asthma. The upper airway component of such conditions is not yet fully identified, and may differ geographically, since CRSwNP can be neutrophilic in the East (30). Absence of asthma may indicate a different pathophysiology, such as the co-existence of CRSsNPs and bronchiectasis, in which alpha 1 anti-trypsin should be measured.

Asthma may precede CRSwNP or parallel the sinonasal disease, however, it may also develop after CRS onset (56). The presence of asthma emphasizes the systemic nature of the underlying pathophysiology and suggests the need for consideration of the disease as a whole. CRS therapy, both medical and surgical, can improve asthma outcomes (118, 119). Further analysis of this relationship is required, with potentially positive effects on both upper and lower airway symptoms the use of specific monoclonal antibodies such as anti-IL5, anti-IL4/13 receptor, and anti-TSLP. The pulmonologist should be encouraged to take an interest in the upper airway, and the ENT surgeon in the lower, including history-taking, examination and specific testing. Good collaboration might help in further resolving the CRS and asthma endotypes, both in an individual patient and in general.

A specific phenotype in which asthma and CRSwNP co-occur in a triad together with hypersensitivity to acetylsalicylic acid, is non-steroidal anti-inflammatory drug (NSAID) exacerbated respiratory disease (N-ERD). It may be considered a type 2 dominated inflammatory airway disorder. N-ERD is slightly more common in females and has an estimated prevalence of 9% in patients with asthma. The pathophysiology is considered an alteration of the cyclo-oxygenase (COX) pathway (2). The CRSwNP in N-ERD patients shows a higher rate of recurrence, and multiple sinus surgeries at younger age (120). Also asthma is more severe with more frequent exacerbations (118). Aspirin desensitization can be considered if an N-ERD patient has insufficient response to CRS treatment and/or insufficient control of asthma symptoms (121). However, discontinuation of this therapy hampers correct long-term evaluation and still questions its actual value (122). A recent retrospective study concluded that nasal polyp eosinophilia, the frequent need of oral corticosteroid courses and a history of recurrent CRSwNP surgery were consistent factors predicting uncontrolled N-ERD (123).

### Outcomes

Real life data and studies may help in understanding the long-term expectations and need for close medical follow-up as we now understand in the CRSwNP phenotype. In a meta-analysis Loftus et al. found a long-term revision rate of ~14 up to 24% (based on different follow-up periods) and retained important risk factors including AFS, AERD, asthma and prior surgery (124). Over a minimal 10-year follow-up Vlaminck et al. retained a revision rate of 26% (34 of 133) stressing the importance of eosinophilic mucin presence on asthma development or aggravation and nasal polyp recurrence (56). The Utah Population Database was queried for Current Procedural Terminology codes for ESS from 1996 to 2016 by Smith et al. reporting an overall revision rate of 30% (*n* = 9,177) over those 20 years (125). Also, the presence of comorbid asthma and allergy were significant predictors of revision surgery (125). In spite of the low number of patients Calus et al. reported a revision rate of 36.8% (14 of 38) over a 12-year period, finding comorbid allergic sensitization and tissue IL-5 levels to be significant predictors (126). The help of digital health technology might be considered as it becomes more apparent some clinical features might be associated with specific inflammatory endotype patterns (127).

## Summary And Outlook

CRS and its treatment are considerably better understood by improved understanding of the immune pathways behind various types of inflammation, in addition to clinical signs and symptoms of the disease. The ENT surgeon can investigate nasal secretions for eosinophilia as a guide to likely T2 inflammation and corticosteroid responsiveness. This is probably most useful in CRSsNP where it is an unexpected finding. Switching from an organ-based to a molecular-based classification in immune-mediated inflammatory diseases helps us to explain the involvement of different organs and the differences among diseases affecting the same organ. Therapeutic consequences are largely based on the responses to anti-cytokine monoclonal antibodies and might better address pathophysiological commonalities across these diseases. An approach based on signature cytokine hubs is likely to yield further insights into etiopathogenesis (128). The prediction of treatment still needs further research, and one of the challenges is co-operation to provide big data on CRS immunopathology related to various treatment outcomes. Patients themselves can participate using mobile data and a VAS score to simply evaluate their upper and lower airway symptoms and quality of life along with the therapy being used.

## Author Contributions

SV drafted the first version. FA and PG edited the manuscript significantly. GS and BL revised the work critically for important intellectual content. All authors made substantial contributions to the design of the work, provided approval for publication of the content, and agreed to be accountable for all aspects of the work.

## Conflict of Interest

The authors declare that the research was conducted in the absence of any commercial or financial relationships that could be construed as a potential conflict of interest.

## Publisher's Note

All claims expressed in this article are solely those of the authors and do not necessarily represent those of their affiliated organizations, or those of the publisher, the editors and the reviewers. Any product that may be evaluated in this article, or claim that may be made by its manufacturer, is not guaranteed or endorsed by the publisher.
